# Role of Connexin 43 in an Inflammatory Model for TMJ Hyperalgesia

**DOI:** 10.3389/fpain.2021.715871

**Published:** 2021-08-03

**Authors:** Fabeeha Ahmed, Md. Rahman, Randall Thompson, David A. Bereiter

**Affiliations:** Department of Diagnostic and Biological Sciences, University of Minnesota School of Dentistry, Minneapolis, MN, United States

**Keywords:** connexins, estrogen status, hyperalgesia, sex differences, temporomandibular joint, trigeminal ganglion

## Abstract

Temporomandibular joint disorders (TMD) consist of a heterogeneous group of conditions that present with pain in the temporomandibular joint (TMJ) region and muscles of mastication. This project assessed the role of connexin 43 (Cx43), a gap junction protein, in the trigeminal ganglion (TG) in an animal model for persistent inflammatory TMJ hyperalgesia. Experiments were performed in male and female rats to determine if sex differences influence the expression and/or function of Cx43 in persistent TMJ hyperalgesia. Intra-TMJ injection of Complete Freund's Adjuvant (CFA) caused a significant increase in Cx43 expression in the TG at 4 days and 10 days post-injection in ovariectomized (OvX) female rats and OvX females treated with estradiol (OvXE), while TG samples in males revealed only marginal increases. Intra-TG injection of interference RNA for Cx43 (siRNA Cx43) 3 days prior to recording, markedly reduced TMJ-evoked masseter muscle electromyographic (MMemg) activity in all CFA-inflamed rats, while activity in sham animals was not affected. Western blot analysis revealed that at 3 days after intra-TG injection of siRNA Cx43 protein levels for Cx43 were significantly reduced in TG samples of all CFA-inflamed rats. Intra-TG injection of the mimetic peptide GAP19, which inhibits Cx43 hemichannel formation, greatly reduced TMJ-evoked MMemg activity in all CFA-inflamed groups, while activity in sham groups was not affected. These results revealed that TMJ inflammation caused a persistent increase in Cx43 protein in the TG in a sex-dependent manner. However, intra-TG blockade of Cx43 by siRNA or by GAP19 significantly reduced TMJ-evoked MMemg activity in both males and females following TMJ inflammation. These results indicated that Cx43 was necessary for enhanced jaw muscle activity after TMJ inflammation in males and females, a result that could not be predicted on the basis of TG expression of Cx43 alone.

## Introduction

Temporomandibular joint disorders (TMD) represent a diverse group of conditions accompanied by pain in the temporomandibular joint (TMJ) region and muscles of mastication. TMD is the most common non-dental orofacial pain condition and is the main reason for TMD patients to seek medical treatment (1, 2). Although routine clinical examinations in TMD typically find little evidence of tissue or nerve damage (3, 4), results from more invasive diagnostic methods such as synovial fluid sampling (5) or jaw muscle microdialysis sampling (6, 7) suggest that TMD is characterized as a persistent mild inflammatory condition. A second prominent feature of TMD is the higher prevalence in women than men (8, 9). Pressure pain thresholds are reportedly lower in female than male TMD patients (10) and vary over the menstrual cycle (11) to further suggest that estrogen status is a key factor for TMD pain in women.

Chronic pain conditions are thought to be driven and maintained by combination of peripheral and central neural mechanisms (12, 13). The TMJ and masticatory muscles are supplied by sensory neurons whose cell bodies lie within the trigeminal ganglion (TG) and dorsal root ganglia of the upper cervical spinal cord (14–16). Results from *in vitro* studies suggest that TMJ nociceptors are sensitized after local inflammation (17) and are further enhanced by estrogen treatment (18). Other studies have shown that ion channels in TG neurons associated with nociception are upregulated by TMJ inflammation and further enhanced by elevated estrogen conditions (19, 20). A key mechanism linking inflammation to sensitization of nociceptors involves activation of satellite glial cells (SGC), a class of non-neuronal cells that surround sensory neurons. SGCs serve a homeostatic function and amplify the effects of local inflammation on the excitability of nociceptors by releasing pro-nociceptive molecules (21–23). Inflammation of the TMJ region activates SGCs in the TG (24–27) resulting in an increase in coupling between SGCs and the formation of gap junctions (28). Connexin 43 (Cx43) is the most common gap junction protein in the TG and is mainly restricted to SGCs (29–32). Although Cx43 expression is regulated in a sexually-dimorphic manner in other tissues (33, 34), no previous studies have determined if sex differences and/or estrogen status alter Cx43 expression and function in an animal model for TMJ hyperalgesia.

The present study also was designed to address the key features of TMD in an animal model. Thus, we used an intra-TMJ injection of Complete Freund's Adjuvant (CFA) at a dose (10 μg) that produces minimal signs of tissue damage (35). Second, we monitored changes in a specific jaw-related muscle behavior, masseter muscle electromyography (MMemg), a signature activity that persists throughout the 10 day observation period following CFA injection. MMemg activity is a valid measure of jaw hyperalgesia since intra-TMJ injection of algesic agents evokes activity in a dose-dependent manner that correlates with pain reports in humans (36). Third, we determined if Cx43 expression and its role in TMJ hyperalgesia are sexually dimorphic and/or are dependent on estrogen status to address the issue that the vast majority of preclinical studies for pain have been conducted in male animals (37, 38).

## Materials and Methods

### General Animal Preparation

A total of 133 adult male, ovariectomized females (OvX) and estradiol-treated OvX female (OvXE) rats (250–350 g, Sprague–Dawley, Harlan, Indianapolis, IN) were used in these experiments. OvX females were purchased commercially and used within 2 weeks of ovariectomy. OvXE rats were injected with estradiol (E2, 30 μg/kg, sc) 1 day prior to processing tissue for immunohistochemical or molecular analyses or for muscle recording. This dose of E2 results in a blood level of E2 consistent with the surge of E2 seen in the proestrous phase of cycling female rats (39). Vaginal lavage samples were taken on the day of the experiment to confirm the estrogen status of females. Samples from OvX rats had mainly small nucleated leukocytes, while samples from OvXE rats had mainly large nucleated epithelial cells consistent with the early diestrous and proestrous stages of the estrous cycle, respectively. Animals were housed in pairs and given free access to food and water. Climate and lighting were controlled (25 ± 2°C, 12:12-h light/dark cycle, light on at 7:00 A.M.). All animal protocols were approved by the Institutional Animal Care and Use Committee of the University of Minnesota (USA) and according to guidelines set by The National Institutes of Health guide for the Care and the Use of Laboratory Animals (PHS Law 99-158, revised 2015).

### Complete Freund's Adjuvant Into TMJ

Rats were anesthetized with 5% isoflurane and the fur overlying the TMJ was shaved. A single dose of CFA (10 μg, 10 μl) was injected into the left TMJ via a 33-gauge needle inserted into the TMJ-capsule (~3 mm in deep) and animals survived for 4 or 10 days prior to tissue collection or muscle recording. Controls received an injection of PBS. All rats received a single dose of carprofen (25 mg/kg, i.p) immediately after the intra-TMJ injection. It is not likely that carprofen affected these results since tissue collection and muscle recording were performed 10 days later.

### Immunohistochemistry

Separate groups of males, OvX and OvXE female rats (sham, 4 day CFA, 10 day CFA, four rats per group) were anesthetized with pentobarbital sodium (50 mg/kg, i.p) and the depth of anesthesia was confirmed by the loss of hindlimb withdrawal reflex. Rats were perfused transcardially with heparinized phosphate buffered saline (PBS) followed by 10% buffered formalin. TGs were removed and postfixed overnight in 10% formalin. Transverse sections (30 μm) were cut on a vibratome and collected in 0.01 M PBS. Free-floating sections were incubated in blocking buffer (PBS, 0.1% Triton X-100, 1% secondary serum) for 1 h and then incubated with anti-mouse primary antibody for glial fibrillary acidic protein (GFAP, Abnova MAB107670, Walnut, CA) and anti-rabbit primary antibody for Cx43 (Cell Signaling 3512, Danvers, MA) at 1:1,000 in PBS with 0.1% Triton X-100 overnight at 4°C. Specificity of the antibody to Cx43 was determined previously (40). Sections were rinsed in PBS (x3) and then incubated with anti-mouse Cy2 secondary antibody (Jackson Immunoresearch 715228151 West Grove, PA) and anti-rabbit Cy5 secondary antibody (Jackson Immunoresearch 711175152 West Grove, PA) at 1:500 in PBS in the dark for 2–3 h. Sections were rinsed in PBS (x3), placed on slides and cover slipped with ProLong Gold with 4,6-diamino-2-phenyindole (DAPI, Invitrogen, Carlsbad, CA). Fluorescent-labeled sections were viewed on a Zeiss LSM 700 confocal microscope at 40X magnification. Images were taken at the level of the junction of the maxillary and mandibular (5–7 images per rat). Staining of Cx43 was corrected for brightness without substraction for background, quantified by densitometry using NIH ImageJ Software and quantified without prior knowledge of treatment. Digital gain settings for Cx43 = 1.5 and for GFAP = 1.0. Statistical analyses of densitometry results were assessed by analysis of variance (ANOVA) and *p* < 0.05 was set as the level of significance without prior knowledge of treatment.

### Real-Time Polymerase Chain Reaction

TGs (four per group) were removed from rats following perfusion with saline and RNA LATER solution (Molecular BioProducts, San Diego, CA). RNA was extracted using the Trizol method (Invitrogen, Carlsbad, CA). cDNA was synthesized using iScript cDNA kit (Bio-Rad, Hercules, CA). RT-PCR was performed in triplicate on 2 μL cDNA with QuantStudio 3 (Applied Biosystems) using iQ SYBRgreen supermix (Bio-Rad). Data was analyzed using the ΔΔCT method using UBC as a reference gene. Primer sets were UBC F-tcgtacctttctcaccacagtatctag, R- gaaaactaagacacctccccatca and CX43 F: 5′-taagtgaaagagaggtgccca-3′ R: 5′-gtggagtaggcttggacctt-3′. 40 cycles were employed at 95°C for 15 s, 59°C for 30 s, and 72°C for 30 s.

### Western Blot

TGs (four per group) were removed after saline perfusion, homogenized, and protein extracted using the Trizol method (Invitrogen, Carlsbad, CA). Protein concentration was determined with bicinchroic acid (BCA) assay (Pierce, Rockford, IL). A protein aliquot of 30 μg was separated on 4–15% polyacrylamide gels (Bio-Rad, Hercules, CA) and transferred to nitrocellulose membrane. Membranes were incubated with Cx43 antibody (3512, Cell Signaling, Danvers, MA), followed by Anti-rabbit IRDye 680 (1:15,000, LI-COR, Lincoln, NE). Proteins were visualized with an Odyssey infrared scanner (LI-COR) and arbitrary optical density was determined. Normalizing controls were utilized by simultaneous staining with glyceraldehyde 3-phosphat dehydrogenase (GAPDH) antibody (1:1,000, WH0002597M1, Sigma, St. Louis, MO) followed by goat anti-mouse IRDye 800 (1:15,000, LI-COR). Protein levels were quantified *via* densitometry using NIH ImageJ Software.

### Interference RNA for Cx43

Animals were anesthetized with pentobarbital sodium (50 mg/kg, i.p) and maintained with isoflurane (1–2%). The fur overlying the scalp was shaved and povidone-iodine was applied before surgery. Lidocaine gel (2%) was applied to scalp wound margins and the body temperature was maintained at 38°C with a heating blanket. The animals were placed in a stereotaxic apparatus and a small hole (3–4 mm) was drilled into the left parietal bone (3.5–4 mm anterior to the auricle and 3–4 mm lateral to the midline). The siRNA solution (600 μg, 200 nL, Stealth RNAi Gja1RSS351267, Invitrogen, Carlsbad, CA) was injected into the left TG 7 days after intra-TMJ injection of CFA *via* a 33-gauge needle inserted through a 26-gauge guide cannula positioned stereotaxically and was kept in position at least 10 min after the injection to minimize leakage. The wound margin was closed with sutures and povidone-iodine solution was applied to the surgical wound area. A single dose of carprofen (25 mg/kg, i.p) was injected in each animal to minimize post-surgical pain. Animals survived for 3 days after siRNA injection (i.e., 10 days after intra-TMJ injection of CFA). Sham controls for CFA received an intra-TMJ injection of PBS only with no further treatment and survived 10 days.

### Masseter Muscle Electromyography Recording

Rats (5–6 rats per group) were anesthetized with urethane (1.5 g/kg) and maintained with supplemental isoflurane (1–2%). The animal was placed in a stereotaxic apparatus and a pair of copper electrodes was implanted in the left masseter muscle (0.12 mm diameter, 5 mm interpolar distance) with a 26-gauge needle. A skin incision was made just above the zygomatic process of the temporal bone and a 26-gauge guide cannula was positioned in the TMJ-capsule (~3 mm in deep). At least 1 h elapsed after cannula placement and before recording. MMemg was recorded under two separate protocols. In the first series following siRNA treatment, MMemg was recorded in response to intra-TMJ injections (PBS, 0.01, 0.1, and 1 mM ATP, 20 μl) delivered *via* a 33-gauge needle inserted through the guide cannula over 30 s in a cumulative dose design at 20 min intervals. In the second series, GAP19 (10 mM, 200–300 nl, Tocris, Minneapolis, MN), a mimetic peptide and specific inhibitor of Cx43 hemichannel formation was injected as a single dose (10 mM, 0.2 μl) into the TG via a 26-gauge guide cannula and a 33-gauge injection cannula 10 min prior to repeated intra-TMJ injections of ATP (1 mM, 20 μl). In both series MMemg was recorded continuously for 6 min for each stimulus period; 3 min prior to each ATP test stimulus to establish the baseline activity and 3 min after test stimulus. The rationale for using ATP as a test stimulus was based on earlier studies demonstrating that ATP can be injected repeatedly without causing tachyphylaxis or sensitization (39).

At the end of the experiment the rat was given a bolus of urethane and perfused transcardially with heparinized PBS and RNase-Away like buffer (60 mL). TGs following MMemg recording sessions were removed and (4 TGs per group, ipsilateral to PBS or CFA injection) were processed for mRNA and protein levels of Cx43. The location of the TG injection site was verified histologically from 1 to 2 rats per group upon removal.

### MMemg Data Analysis

MMemg activity was sampled at 1,000 Hz, amplified (×10 k), filtered (bandwidth 300–3,000 Hz), displayed and stored online for analyses. EMG activity was sampled continuously for 6 min, for 3 min prior to each TMJ stimulus and for 3 min after the stimulation was applied. Baseline activity was quantified as the total area under the curve (Total MMemg) for the 3 min epoch (μV-s per 3 min) sampled immediately prior to stimulation. TMJ-evoked MMemg activity was calculated as AUC post-ATP injection minus the baseline.

### Statistical Analyses

**Figure 1:**
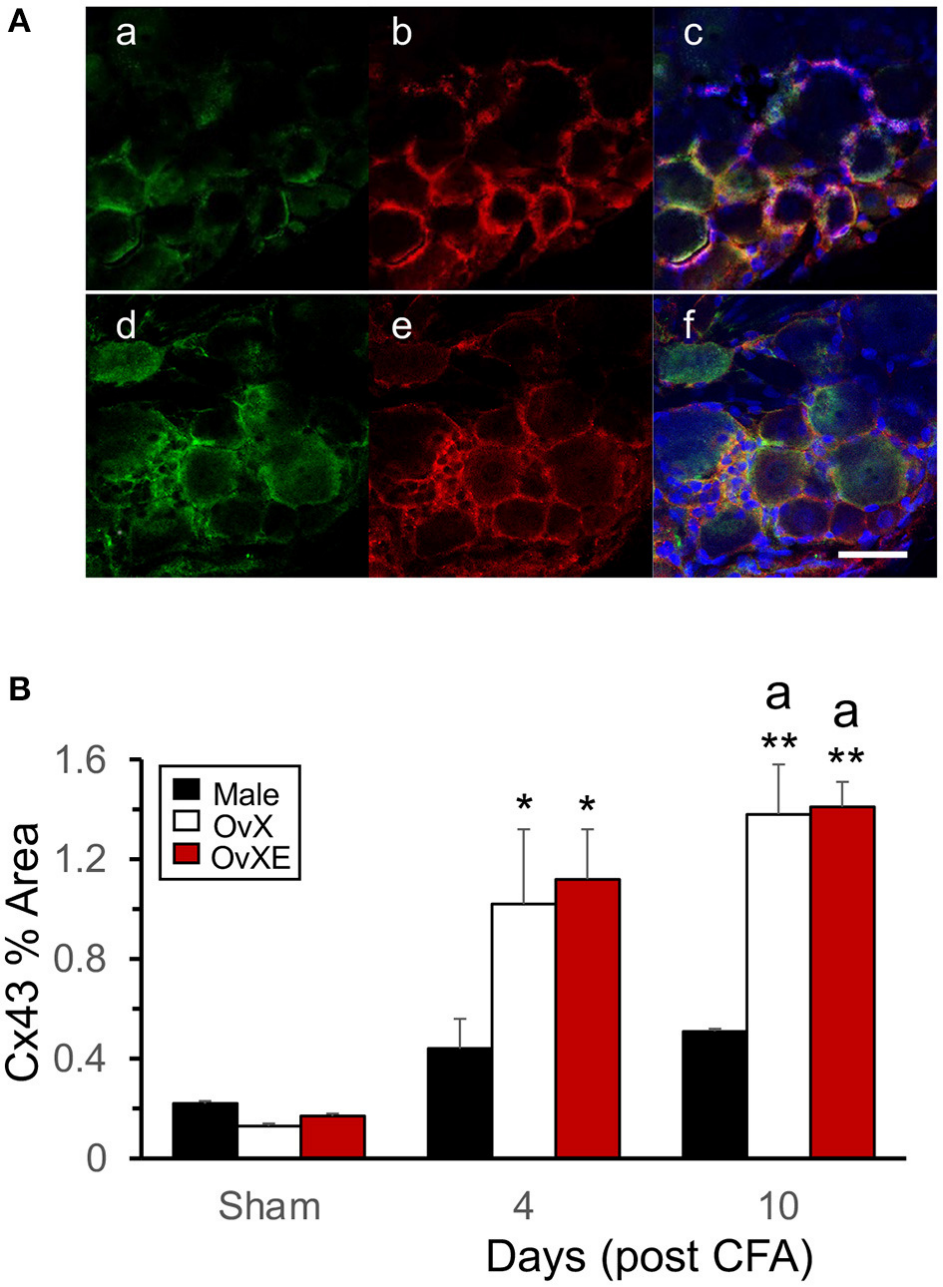
**(A)** Expression of glial fibrillary acidic protein (GFAP) and connexin43 (Cx43) in the trigeminal ganglion of an OvXE (a–c) and male rat (d–f) 10 days after intra-TMJ injection of CFA. Scale = 30 μm. **(B)** Densitometry values expressed as percentage of positive area for Cx43 in male, OvX and OvXE rats under sham conditions and at 4 and 10 days after intra-TMJ injection of CFA based on an average of immunostaining as shown by the examples in panel **(A)**. *p < 0.05, **p < 0.01 vs. sham; ^a^*p* < 0.05 vs. males. Sample size = 4 rats per group, average of 5–7 images per rat.

**Figure 2:**
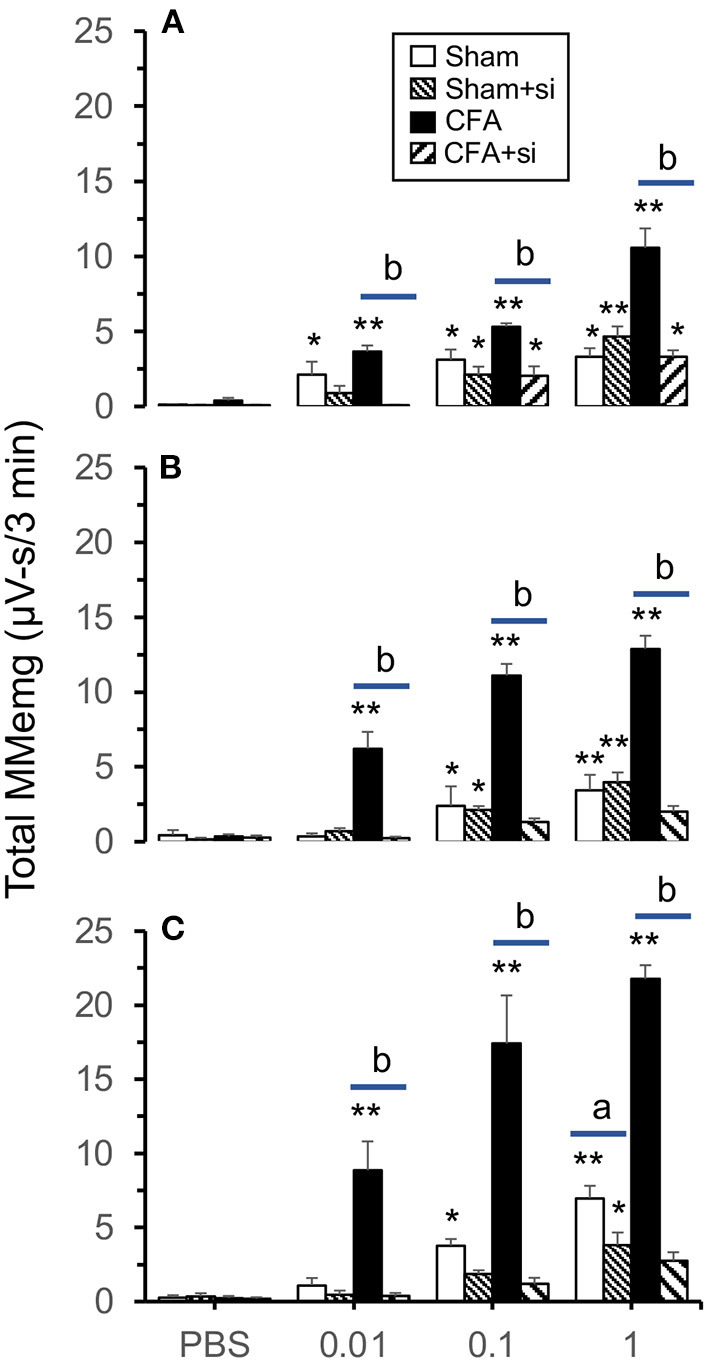
siRNA for Cx43 inhibits intra-TMJ ATP-evoked MMemg activity in **(A)** male, **(B)** OvX and **(C)** OvXE females treated with CFA 10 days prior to recording. Note that responses to TMJ stimuli in sham (PBS-injected) rats were not affected. *p < 0.05, **p < 0.01 vs. PBS stimulation; ^a^*p* < 0.05, ^b^*p* < 0.01 siRNA treated vs. untreated rats. Sample size = 5–6 rats per treatment group.

**Figure 3:**
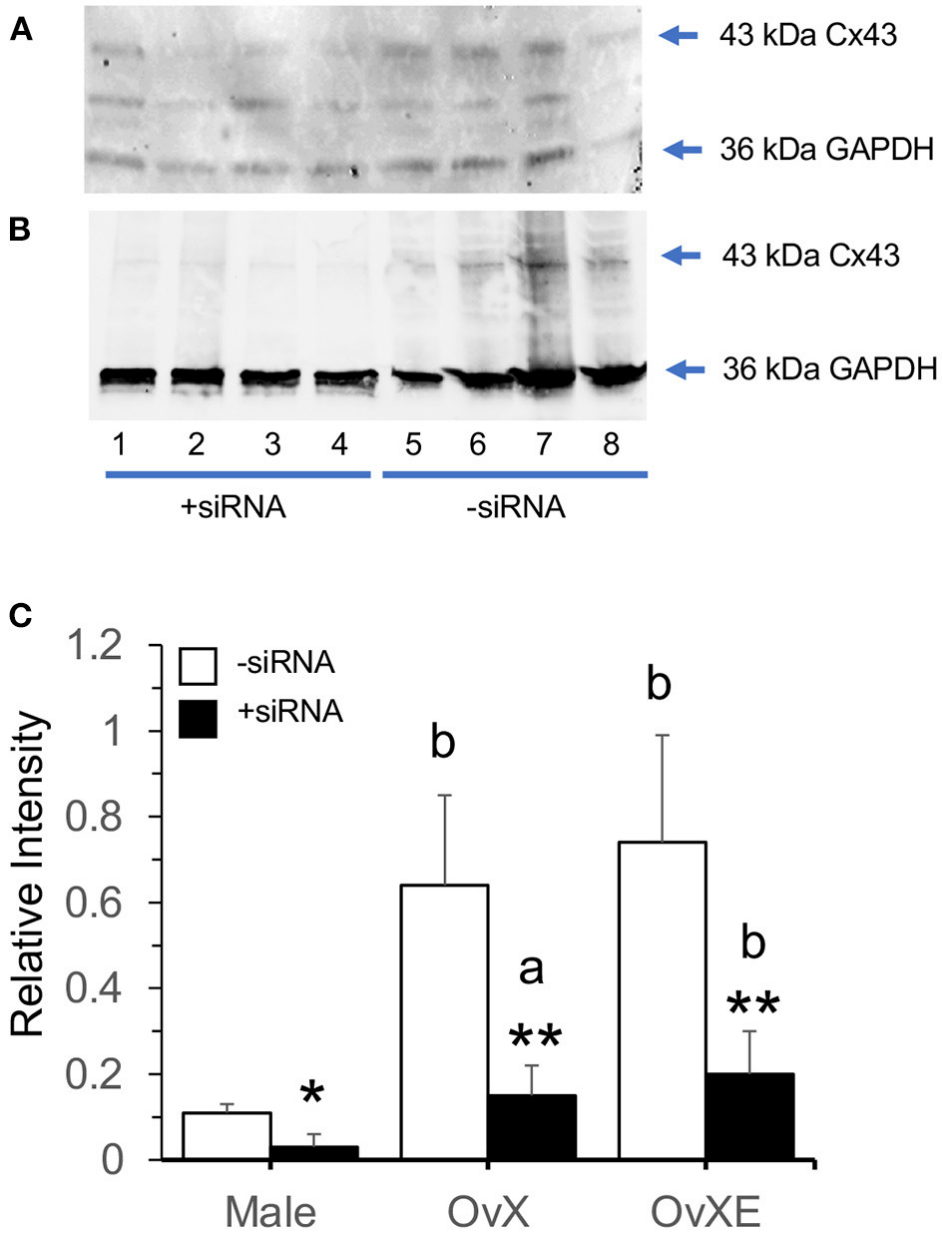
Western blots for Cx43 of TG tissue from OvXE **(A)** and male rats **(B)** at 10 days after CFA and treated with siRNA for Cx43 or with PBS by intra-TG injection 3 days prior to tissue collection. **(C)** Summary of the effects of siRNA for Cx43 on protein levels in the TG of male, OvX, and OvXE females. *p < 0.05; **p < 0.01 vs sham controls; ^a^*p* < 0.05, ^b^*p* < 0.01 vs males vs. sham controls. Sample size = 4 rats per group.

**Figure 4:**
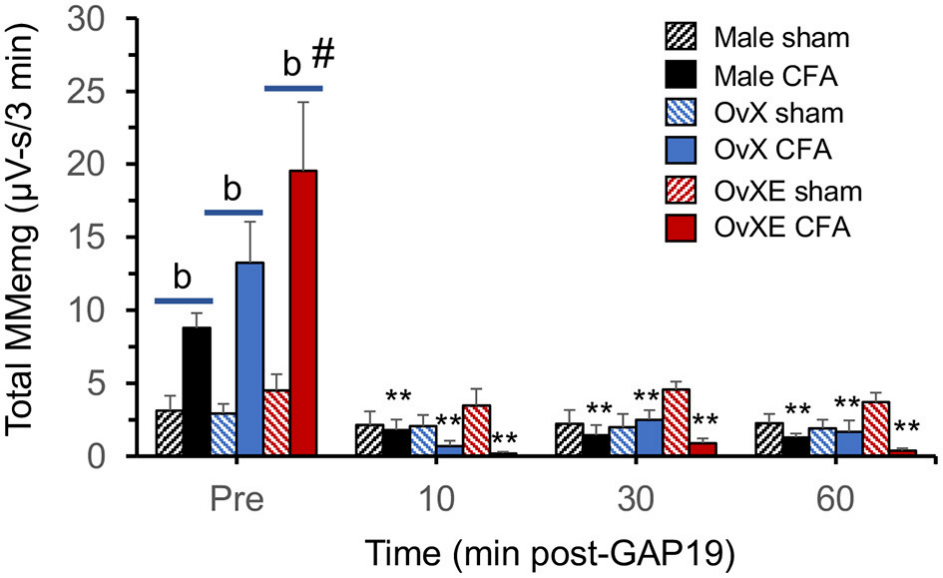
Acute microinjection of GAP19 into the TG reduces the enhanced TMJ-evoked MMemg activity of 10 day CFA-treated males, OvX and OvXE females, while responses in sham rats were not affected. **p < 0.01 vs. pre-injection response; ^b^*p* < 0.01 sham vs. CFA groups; ^#^*p* < 0.01 vs. all other groups. Sample size = 5 rats per group.

Densitometry was assessed from 5 to 7 TG sections per rat (4 rats per treatment group) and expressed as average percent positive area (Figure 1). Sections were analyzed without prior knowledge of treatment. Values were compared by one-way ANOVA and individual group differences assessed by Neuman–Keuls. Total MMemg activity was assessed by three-way ANOVA and corrected for repeated measures on one factor (5–6 rats per group; Figure 2). Significant treatment effects were assessed by Newman–Keuls after ANOVA. The data were presented as mean ± SEM and the significant level set at *p* < 0.05. Based on results from previous studies (41, 42), it was calculated that a sample size of *n* = 5 per treatment group would provide 80% power at *p* < 0.05. Experiments were performed on sham and TMJ-inflamed rats selected in random order. Western blots were performed on TG samples collected from four rats per treatment group (Figure 3). Values were log transformed to reduce error variance and then compared by two-way ANOVA and between group differences assessed by Neuman–Keuls after ANOVA. Total MMemg activity was assessed by three-way ANOVA and corrected for repeated measures on one factor (Figure 4). Significant treatment effects were assessed by Newman–Keuls after ANOVA and included 5–6 rats per treatment group. The data were presented as mean ± SEM and the significance level set at *p* < 0.05. Experiments were performed on sham and TMJ-inflamed rats selected in random order.

## Results

### Immunohistochemistry

Glial fibrillary acidic protein (GFAP) and Cx43 were often co-localized and appeared as stained elements surrounding small and moderate diameter TG neurons of TMJ-inflamed OvXE rats (Figures 1Aa–c) and male rats (Figures 1Ad–f). Figure 1B summarizes the percentage of Cx43 stained area in the TG of sham animals which was very low for males and females. By contrast, Cx43 displayed a marked and sex-dependent increase in Cx43 area at 4 days and 10 days after CFA [*F*_(8,27)_ = 7.01, *p* < 0.001]. Both OvX and OvXE groups displayed significant (*p* < 0.01) and similar increases in Cx43 staining after CFA, while Cx43 staining in CFA-treated males was not statistically different from sham males (*p* < 0.1).

### MMemg and siRNA Cx43

To determine if TG expression of Cx43 altered TMJ-evoked MMemg activity, siRNA for Cx43 was microinjected into the left TG 3 days prior to the recording session. As seen in Figure 2A, sham males displayed small but significant increases in ATP-evoked MMemg activity [F_(3,51)_ = 7.45, *p* < 0.001] that were similar after siRNA knockdown of Cx43 [F_(3,51)_ = 13.7, *p* < 0.001]. By contrast, CFA-treated males displayed significant increases in ATP-evoked MMemg activity in the absence of siRNA [F_(3,51)_ = 62.9, *p* < 0.001] and much smaller after siRNA [F_(3,51)_ = 10.5, *p* < 0.001]. Treatment main effects revealed that siRNA reduced the evoked MMemg activity compared to rats without siRNA treatment [F_(3,17)_ = 26.84, *p* < 0.001]. Figure 2B revealed that OvX sham females displayed small but significant increases in ATP-evoked MMemg activity [F_(3,51)_ = 7.66, *p* < 0.001] that were similar siRNA knockdown of Cx43 [F_(3,51)_ = 9.63, *p* < 0.001]. CFA-treated OvX females (Figures 2B, 3B) displayed large ATP-evoked MMemg responses [F_(3,51)_ = 104, *p* < 0.001] that were completely prevented by siRNA treatment [F_(3,51)_ = 2.98, *p* > 0.1]. Overall treatment main effects revealed that siRNA reduced the ATP-evoked MMemg responses in OvX rats compared to OvX rats without siRNA treatment [F_(3,17)_ = 64.13, *p* < 0.001]. Figure 2C revealed that OvXE sham females displayed large increases in ATP-evoked MMemg activity [F_(3,51)_ = 10.2, *p* < 0.001] that were marginally reduced by siRNA knockdown of Cx43 [F_(3,51)_ = 2.89, *p* < 0.05]. CFA-treated OvXE females displayed the greatest ATP-evoked MMemg responses [F_(3,51)_ = 100, *p* < 0.001] and were completely prevented by siRNA treatment [F_(3,51)_ = 1.79, *p* > 0.1]. Overall treatment main effects indicated that intra-TG siRNA treatment greatly reduced evoked MMemg activity compared to rats without siRNA treatment [F_(3,17)_ = 69.64, *p* < 0.001]. RT-PCR analyses of TG samples revealed no significant sex differences for Cx43 among siRNA-injected sham animals (ΔCT: male = −5.37 ± 2.25; OvX = −5.58 ± 3.7; OvXE = −5.58 ± 1.15, mean ± SD) or at 10 days after CFA (ΔCT: male = −6.06 ± 0.83; OvX = −6.92 ± 5.31; OvXE = −4.53 ± 1.8, mean ± SD). Figures 3A,B displays the western blot for males and OvXE females at 10 days post-CFA, respectively, with and without siRNA knockdown of Cx43. The results for western blots for all groups are summarized in Figure 3C revealing that siRNA for Cx43 significantly reduced TG expression of Cx43 in both males and females [F_(1,18)_ = 10.11, *p* < 0.001].

### MMemg and Pharmacological Blockade of Cx43 Formation by GAP19

To determine if acute blockade of Cx43-dependent hemichannel formation affected ATP-evoked MMemg responses, the peptide mimetic inhibitor of Cx43, GAP19, was microinjected into the left TG of sham male, OvX and OvXE rats and in rats at 10 days after CFA treatment. As seen in Figure 4, CFA-induced enhancement of TMJ-evoked MMemg activity was significant for males and female groups [overall response main effects F_(3,72)_ = 44.19, *p* < 0.001]. GAP19 injection did not significantly affect the ATP-evoked MMemg responses in sham males, OvX or OvXE females [F_(3,72)_ = <1.0, *p* > 0.1]. By contrast, ATP-evoked responses in CFA-treated males [F_(3,72)_ = 8.55, *p* < 0.001], OvX females [F_(3,72)_ = 22.2, *p* < 0.001] and OvXE females [F_(3,72)_ = 58.7, *p* < 0.001] all displayed marked decreases in evoked MMemg activity following GAP19 administration.

## Discussion

These results revealed a significant increase in Cx43 expression in the TG of OvX and OvXE females that persisted for at least 10 days after mild inflammation of the TMJ, while Cx43 expression in males displayed only marginal increases. Two different approaches were used to assess the functional contributions of Cx43 to TMJ-evoked hyperalgesia. First, small interference mRNA for Cx43 was injected into the TG to silence Cx43 expression in sham and 10 day CFA-treated rats. This resulted in a significant reduction in TMJ-evoked MMemg activity in males and both female groups after TMJ inflammation, but not in sham animals, that was matched by a corresponding decrease in Cx43 protein in TG samples. Second, the mimetic peptide, GAP19, a specific inhibitor of hemichannel formation in nervous tissue (43), was injected acutely into the TG of sham and CFA-inflamed rats. This approach also caused a marked decrease in TMJ-evoked MMemg activity in all CFA-treated animal groups, while evoked activity in sham rats was not affected.

Despite numerous preclinical studies directed at understanding the underlying mechanisms for TMJ hyperalgesia, little progress has been made in developing new pharmacological treatments that are specific for TMD pain (1, 44, 45). Several reasons may contribute to this apparent lack of progress; however, one limitation may be the mismatch between the features of animal models for TMJ nociception and the clinical signs in TMD patients. The present study was designed to minimize these mismatches. TMD patients display few overt signs of tissue damage or inflammation yet often present with fluctuating bouts of pain in a non-progressive manner (46–48). By contrast, rodent models for TMJ hyperalgesia often involve treatments that cause significant tissue damage. Indeed, an intra-TMJ injection of even a dose of CFA as low as 10 μg is sufficient to elevate TMJ tissue levels of proinflammatory cytokines and to increase meal duration in rats (35), while CFA doses of 25 μg or greater cause soft tissue damage and progressive bone erosion (49, 50). A second feature of a valid model for TMJ hyperalgesia is the ability to monitor a surrogate measure of TMJ hyperalgesia. The present study monitored MMemg activity which is a behavior that specifically assesses jaw function and persists throughout the 10 day observation period following CFA treatment. Other direct measures of TMJ hyperalgesia in awake rats such as a decrease in gnawing behavior (51, 52) or bite force (53, 54) and an increase in grimace scale values (52) are seen following intra-TMJ injection of CFA; however, changes in these behaviors are transient and often only a few days. Increased meal duration has been shown to persist for many days after CFA in rats (55); however, this required much larger doses of CFA than that used in the present study (250 μg vs. 10 μg). A third feature of the present model was the comparison of results in males vs. females under high and low estrogen status. Despite the higher prevalence of TMD in females than males (8, 9), few preclinical studies have directly compared responses of males and females for TMJ hyperalgesia. The rationale for using ATP as a test stimulus to evoke MMemg activity was based on two lines of evidence. First, earlier we determined that a 1 mM concentration of ATP reliably evoked trigeminal brainstem activity and could be injected repeatedly at 20 min intervals within the TMJ without causing persistent sensitization or tachyphylaxis (56) and secondly, that ATP is a normal constituent of synovial fluid and evokes increases in pain intensity in a dose-dependent manner (57).

A critical unresolved issue in chronic TMD is the relative contribution of peripheral sensitization of nociceptors in driving long-term changes in central neural processing. Although synovial fluid sampling in TMD patients reveal increased levels of pro-nociceptive molecules such as serotonin and glutamate, the levels of molecules and the magnitude of pain intensity are not well-correlated (5). It is widely accepted that both peripheral and central neural mechanisms contribute to most chronic pain conditions (12, 13). The inhibitory effects of local knockdown of Cx43 within the TG by siRNA or by acute blockade of Cx43-dependent hemichannel formation on TMJ-evoked MMemg activity suggest that Cx43 contributes to a persistent peripheral driving force to enhance TMJ hyperalgesia after inflammation. Cx43 is the most abundant of several connexins expressed in the TG (30). Previous studies have reported that Cx43 expression in the TG was elevated at 8–10 days after trigeminal nerve injury (58, 59), at 3 days after tooth pulp inflammation (31) and 1 day after TMJ inflammation (32) in male rats. Garrett and Durham (30) reported increases in Cx26, Cx36, and Cx40 at 3 days after TMJ inflammation in male rats with no increase in Cx43 in the TG. Interestingly, we also found only marginal increases in Cx43 in the TG of male rats at 4 and 10 days after CFA, while marked increases in Cx43 were seen for OvX and OvXE female groups. This finding underscores the necessity of performing preclinical studies on female as well as male animals. There may be several reasons for the apparent mismatch between the marginal increase in Cx43 expression in the TG after TMJ inflammation and the significant reduction in TMJ-evoked MMemg activity and the reduction in Cx43 protein after siRNA in males. First, we cannot exclude that testosterone offers some level of protection to developing TMJ hyperalgesia after inflammation as has been suggested previously (60–62). Second, TG neurons that drive the TMJ-evoked MMemg activity in males may be more sensitive to increases in Cx43 compared to females and may require only minimal changes to be effective. Third, estrogen reduces the degradation of Cx43 in cardiac tissue (63) and thus, due to its rapid turnover (64), Cx43 protein may remain elevated for longer times in females. The short half-life of Cx43 may also explain the lack of change in mRNA at 3 days after siRNA injection. Fourth, it is possible that post-translation requirements such as the rate of phosphorylation may be different in males and females (64). Indeed, earlier we reported that estrogen status and inflammation interact through kinase-dependent mechanisms to enhance TMJ hyperalgesia (65).

The present study used a model for TMJ hyperalgesia that addressed several of the features typically seen in TMD patients to conclude that Cx43 plays a critical role in maintaining TMJ homeostasis after low levels of inflammation. Inhibition of Cx43 by siRNA or by acute blockade of Cx43-dependent hemichannel formation by GAP19 caused a significant decrease on TMJ-evoked MMemg, a valid surrogate measure of TMJ hyperalgesia, in both males and females. Lastly, we found similar changes in Cx43 expression in the TG and inhibitoion of response magnitudes to siRNA or GAP19 on TMJ-evoked MMemg in OvX and OvXE females. These results suggest that that estrogen status alone is not a significant determinant of the influence of Cx43 on TMJ hyperalgesia. However, the fact that inhibition of Cx43 function significantly reduced the effects on TMJ hyperalgesia in both males and females suggest that approaches that target Cx43 may be a novel therapeutic approach to manage TMD pain.

## Data Availability Statement

The original contributions presented in the study are included in the article/supplementary material, further inquiries can be directed to the corresponding author/s.

## Ethics Statement

The animal study was reviewed and approved by IACUC, University of Minnesota.

## Author Contributions

FA, MR, and RT performed experiments and collected and analyzed data. MR and DB designed experiments. DB analyzed data and prepared the manuscript. All authors contributed to the article and approved the submitted version.

## Conflict of Interest

The authors declare that the research was conducted in the absence of any commercial or financial relationships that could be construed as a potential conflict of interest.

## Publisher's Note

All claims expressed in this article are solely those of the authors and do not necessarily represent those of their affiliated organizations, or those of the publisher, the editors and the reviewers. Any product that may be evaluated in this article, or claim that may be made by its manufacturer, is not guaranteed or endorsed by the publisher.
